# Satellite derived offshore migratory movements of southern right whales (*Eubalaena australis*) from Australian and New Zealand wintering grounds

**DOI:** 10.1371/journal.pone.0231577

**Published:** 2020-05-07

**Authors:** Alice I. Mackay, Frédéric Bailleul, Emma L. Carroll, Virginia Andrews-Goff, C. Scott Baker, John Bannister, Laura Boren, Krisa Carlyon, David M. Donnelly, Michael Double, Simon D. Goldsworthy, Robert Harcourt, Dirk Holman, Andrew Lowther, Guido J. Parra, Simon J. Childerhouse

**Affiliations:** 1 South Australian Research and Development Institute, Primary Industries and Regions South Australia, Adelaide, South Australia, Australia; 2 School of Biological Sciences, University of Adelaide, Adelaide, South Australia, Australia; 3 School of Biological Sciences, University of Auckland, Auckland, New Zealand; 4 Sea Mammal Research Unit, Scottish Oceans Institute, University of St Andrews, Fife, Scotland; 5 Australian Antarctic Division, Australian Marine Mammal Centre, Kingston, Tasmania, Australia; 6 Hatfield Marine Science Center, Newport, Oregon, United States of America; 7 Deceased, Western Australian Museum, Welshpool DC, Western Australia, Australia; 8 New Zealand Department of Conservation, Wellington, New Zealand; 9 Marine Conservation Program, Tasmanian Department of Primary Industries, Parks, Water and Environment, Hobart, Tasmania, Australia; 10 Killer Whales Australia, Box Hill, South Victoria, Australia; 11 Department of Biological Sciences, Macquarie University, Sydney, New South Wales, Australia; 12 Department of Environment & Water, Port Lincoln, South Australia, Australia; 13 Norwegian Polar Institute, Tromsø, Norway; 14 Cetacean Ecology, Behaviour and Evolution Lab, Flinders University, Adelaide, South Australia, Australia; 15 Blue Planet Marine, Nelson, New Zealand; Institute of Deep-sea Science and Engineering, Chinese Academy of Sciences, CHINA

## Abstract

Southern right whales (*Eubalaena australis*) migrate between Austral-winter calving and socialising grounds to offshore mid- to high latitude Austral-summer feeding grounds. In Australasia, winter calving grounds used by southern right whales extend from Western Australia across southern Australia to the New Zealand sub-Antarctic Islands. During the Austral-summer these whales are thought to migrate away from coastal waters to feed, but the location of these feeding grounds is only inferred from historical whaling data. We present new information on the satellite derived offshore migratory movements of six southern right whales from Australasian wintering grounds. Two whales were tagged at the Auckland Islands, New Zealand, and the remaining four at Australian wintering grounds, one at Pirates Bay, Tasmania, and three at Head of Bight, South Australia. The six whales were tracked for an average of 78.5 days (range: 29 to 150) with average individual distance of 38 km per day (range: 20 to 61 km). The length of individually derived tracks ranged from 645–6,381 km. Three likely foraging grounds were identified: south-west Western Australia, the Subtropical Front, and Antarctic waters, with the Subtropical Front appearing to be a feeding ground for both New Zealand and Australian southern right whales. In contrast, the individual tagged in Tasmania, from a sub-population that is not showing evidence of post-whaling recovery, displayed a distinct movement pattern to much higher latitude waters, potentially reflecting a different foraging strategy. Variable population growth rates between wintering grounds in Australasia could reflect fidelity to different quality feeding grounds. Unlike some species of baleen whale populations that show movement along migratory corridors, the new satellite tracking data presented here indicate variability in the migratory pathways taken by southern right whales from Australia and New Zealand, as well as differences in potential Austral summer foraging grounds.

## Introduction

Baleen whales undertake annual migrations between productive feeding grounds and sheltered calving grounds [1–3]. Maternally transmitted fidelity to feeding grounds has been proposed for several species including humpback whales (*Megaptera novaeangliae)* ([4,5] southern right whales (*Eubalaena australis*) [6–8], and North Pacific gray whales (*Eschrichtius robustus*) [9].

Southern right whales have a circumpolar distribution between latitude 16°S and 65°S, albeit with a discontinuation between New Zealand and Chile (90°W to 180°W). The species typically migrates between mid-latitude, Austral-winter calving grounds and offshore mid- to high latitude Austral-summer feeding grounds [10]. In Australasia, contemporary wintering grounds extend from Western Australia across southern Australia to the New Zealand sub-Antarctic Auckland and Campbell Islands, with occasional sightings up the eastern coast of Australia, around the North and South Islands of New Zealand (mainland New Zealand) and the Australian sub-Antarctic Macquarie Island [10–15].

The species was heavily exploited across its range, with up to 66,000 whales taken between 1790 and 1980 from both shore and pelagic whaling grounds around New Zealand and southeastern Australia [16]. Whaling was also conducted from West and South Australia, but the number killed is unknown due to poor historical records [17]. In Australia, the post-whaling recovery rate of southern right whales is markedly higher in the southwest (i.e., West Australia and western parts of South Australia) than in the southeast (i.e., eastern parts of South Australia, Tasmania, Victoria and New South Wales). This difference, as well as evidence of genetic substructure, has led to southern right whales in Australia being considered as two sub-populations or management units—southwest (SWA) and the southeast (SEA) Australia with the boundary between the two sub-populations at approximately 140°E [18].

Abundance estimates for the SWA sub-population in 2014 was 2,300 [19] compared to an estimate of 257 individuals in the same year for the SEA [20]. The main wintering area in New Zealand is at the sub-Antarctic Auckland Island with an estimated population size of 2,139 whales in 2009 [21]. Both the SWA sub-population and New Zealand sub-population are estimated to be recovering at approximately 6–7% per annum [21, 22]. The SWA sub-population is presently expanding into former calving grounds [23] and in New Zealand, whales are returning to former wintering grounds around the mainland [13]. There is no evidence of any significant recovery for the SEA sub-population.

Female southern right whales show strong fidelity to winter calving grounds [7–8, 11–12, 24], with males showing a lower degree of fidelity [7, 12, 18, 25]. There is also growing evidence, based on both genetic and stable isotope data, of maternally directed fidelity to summer feeding grounds [6,7]. Southern right whale breeding success in both Argentina and Brazil has been correlated with changes in sea surface temperature at feeding grounds in the South Atlantic [26,27], therefore fidelity to feeding grounds that may be sub-optimal could limit population recovery.

Individual southern right whales from the same breeding populations have been found to show different isotopic prey signatures [6], and to utilise a range of summer foraging grounds within the same ocean basin [28,29]. Historical whaling data show southern right whales were captured in the region of the Subtropical Front (STF) south of Australia during the Austral summer months [30], and north and east of New Zealand [31,32]. The STF, which typically occurs between latitudes 39°–42°S, is a continuous feature that lies within the Southern Tropical Convergence (STC) and is characterized by an area of elevated primary productivity [33, 34]. Such oceanographic fronts in the Southern Ocean are important foraging areas for a range of marine predators [35].

The deployment of satellite telemetry devices has provided critical information on the distribution, migration and seasonal movements of many species of large whales [28–29, 36–40]. Such information is required to assess and manage potential impacts of anthropogenic activities on highly migratory species and to identify potential drivers that may be hindering recovery [10]. This study applies satellite telemetry to obtain novel information on migratory pathways of southern right whales from Australian and New Zealand wintering grounds, and to identify potential feeding grounds.

## Methods

### Ethic statement

Deployment of satellite tags and collection of biopsy samples were conducted in strict accordance with the approvals and conditions from relevant Animal Ethics Committees and State and Commonwealth research permits: *Auckland Islands*, *New Zealand*—Australian Antarctic Division (EPBC Permit 2007–007; AAEC approval 2941-09/10) and the Department of Conservation (Marine Mammal Research Permit SO-2571-MAR; Animal Ethics Committee approval AEC 195). Biopsy samples were collected under a Marine Mammal Research Permit (RNW/HO/2009/03) provided to Prof. C. S. Baker by the New Zealand Department of Conservation and under a University of Auckland Animal Ethic Protocol (AEC/02/2005/R334). *Tasmania*—Department of Primary Industries, Parks, Water and Environment (AEC approval 29/2009-10; Permit to Take Threatened Fauna for Scientific Purposes TFA10106). *Head of Bight*, *Australia*—Primary Industries and Regions South Australia (PIRSA) Animal Ethics Approval and under the following permits: PIRSA Fisheries Exemption (ME9902712), Department of Environment Water and Natural Resources (DEWNR) Permit and Licence to Undertake Scientific Research (A24684-12), EPBC Cetacean Permit (20014–0004), Access to Biological Resources in a Commonwealth Area for Non-commercial Purposes (AU-COM2014-248), Approval for Activity in Commonwealth Marine Reserve (CMR-14-000196) and DEWNR Marine Parks Permit (MO00024-2).

### Satellite tag settings and deployment

Satellite tags deployed at Auckland Islands and Tasmania comprised Spot 5 (location only) satellite transmitters (Wildlife Computers Ltd, Redmond, Washington, USA) encased in an implantable housing designed by the Australian Antarctic Division (AAD, Hobart, Tasmania, Australia) in conjunction with Sirtrack Ltd (Havelock North, New Zealand). The stainless-steel cylindrical housing plus anchor section is 320 mm in length, and upon implantation the tag penetrates the skin and blubber where it is retained by the spring-loaded (articulated) anchor and passively deployed petals. Tags deployed at Head of Bight were not articulated and were fitted with a stainless-steel collar to reinforce the bolt that connected the anchor to the cylindrical electronics housing. Tags were sterilised with ethylene oxide (Auckland Islands and Tasmania) or methylated spirits and chlorhexidine (Head of Bight) prior to deployment. On immersion, the salt-water switch is activated, and the tag location is transmitted through the ARGOS satellite network. Tags were deployed using a modified pneumatic line-thrower (ARTS; Air-Rocket-Transmitter-System—see Heidi-Jorgenson *et al*., 2001 for details) set at 7.5–11 bar of pressure and fired at distances ranging from 2–8 m from the individual. Satellite tags were programmed with a duty cycle of 6 hr on and 18 hr off (Auckland Islands), 4 hr on and 8 hr off (Tasmania) and 3 hr on and 3 hrs off (Head of Bight). Transmitters were aimed to be deployed at the highest point on the whale’s back, close to the dorsal midline between the pectoral fins and slightly forward of where the dorsal fin would be (if the species had one), to minimise physiological responses to implantation and ensure good antenna exposure.

### Satellite telemetry analysis

**Fig 1:**
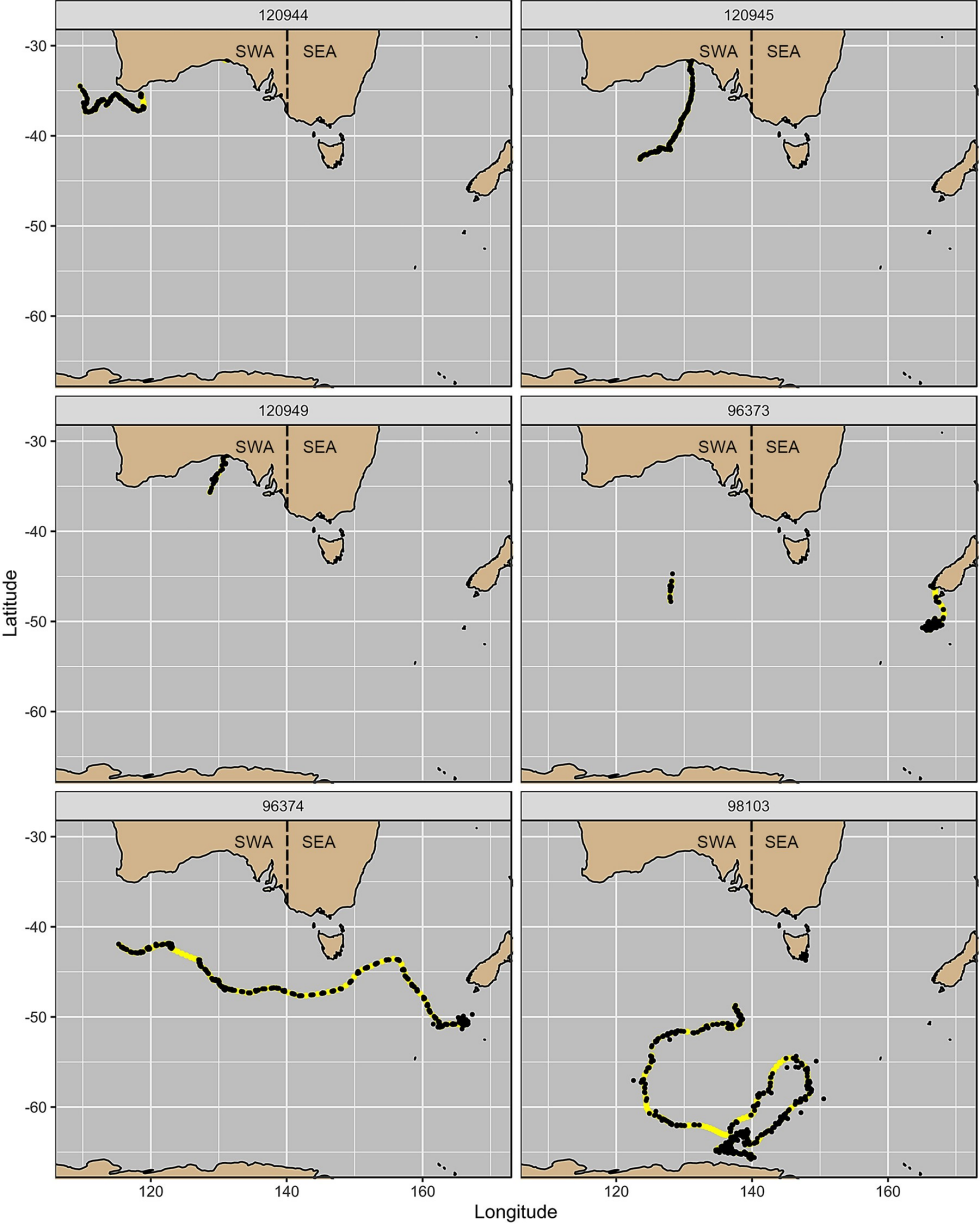
Raw Argos location data transmitted for six southern right whales equipped with satellite telemetry (black dots) and reconstructed tracks after raw location data were interpolated and filtered (yellow lines). Individuals 96373 and 96374 were tagged at the Auckland Islands, New Zealand. Individual 98103 was tagged at Pirates Bay, Tasmania, Australia. Individuals 120944, 120945 and 120949 were tagged at Head of Bight, Australia. The horizontal dashed line over continental Australia represents the approximate delineation between the proposed Australian sub-populations; southwest Australia (SWA) and southeast Australia (SEA).

Satellite telemetry positions were filtered using the class-based location quality estimates provided by Argos (based on the precision and accuracy of location estimates) from correlated random walk modelling within a state-space framework (DCRWS). The errors in satellite-derived locations provided by Argos were incorporated into estimates of likely position, and their precision was estimated using either Bayesian [41, 42] or maximum likelihood methods [43]. State-space models allow unobserved states and biological parameters to be estimated from location estimate data (i.e. foraging/resting vs. travelling). The ARGOS-derived locations were observed irregularly through time (sometimes with a large gap between successive location), which imposes an artificial perspective on the movement processes. State space models account for these features of the data and allow filtering/interpolating spatial positions [41]. Models were fitted using JAGS 3.1.0 (Just Another Gibbs Sampler, http://martynplummer.wordpress.com; http://mcmc-jags.sourceforge.net) accessed from R (R Core Team 2015) using the package ‘bsam’ [44]. The model estimated two locations per day (Fig 1) and two Markov chains with a total of 50,000 simulations were computed, only keeping one out ten samples to minimise sample autocorrelation. The analyses assumes a time-step of two hours and generate 25,000 samples per chain for each position. The model also classified locations into two behavioural modes based on mean turning angles and autocorrelation in speed and direction: transiting (mode 1) and Area Restricted Search (ARS, mode 2). The two behavioural modes estimated from the DCRWS model were delineated by adopting cut-offs of the mean estimates at 1.25 and 1.75; mean estimates below 1.25 were considered to represent transiting and mean estimates above 1.75 were considered to represent foraging / ARS. Mean estimates between 1.25 and 1.75 were treated as uncertain, i.e. there was insufficient information to distinguish between the behaviours in these cases [45].

For each interpolated location the following environmental data were extracted using the data that was closest in time to the location transmission date: sea surface temperature (SST), current (e.g., flow) magnitude and current direction. SST data (1/4° x 1/4° on a Cartesian grid) were read from OISST (http://www.ngdc.noaa.gov/oisst) and current data (1/4° x 1/4° on a Cartesian grid) were read from AVISO current data (http://www.aviso.altimetry.fr/en/data/products/sea-surface-height-products/global/madt.html). For each individual track, the position of three major ocean frontal regions in the study area were approximated separately for each track based on the average SST that occurred during the transmission period of each individual tag, and the reported temperature ranges of these frontal systems; 11° and 12°C for the Subtropical front, 7° and 8°C for the sub-Antarctic front and 4° and 5°C for the polar front [46]). Fronts form boundaries between distinct water masses due to sharp gradients in temperature and / or salinity. The trajectories of marine animals are a combination of the individual’s voluntary motion (i.e., swimming) and its transportation by oceanic currents (i.e., drift). Therefore, the observed velocity of the animal is the sum of the individual’s swimming velocity and the velocity of the current. To assess the effect of current on animal trajectories, we removed the current component from the observed tracks and ‘reconstructed’ current-corrected tracks (i.e., the trajectory that the individual would have followed in a motionless ocean [47].).

## Results

### Satellite tagging—Large scale movement patterns

In total, 16 satellite tags were deployed on adult right whales. Of these, six tags were deployed on unaccompanied adult right whales at the Auckland Islands, New Zealand (50.5°S 166.3°E) between 24 July and 2 August 2009 and one tag was deployed on a sub-adult at Pirates Bay, Tasmania (43.2°S 147.9°E) in October 2010. Nine tags were also deployed on adult right whales (eight accompanied females and one unaccompanied whale) at Head of Bight, South Australia (31.5°S 131.1°E) on 6 and 7 September 2015. Tag performance was highly variable. Three of the six tags deployed at the Auckland Islands (AI) ceased transmitting before the individuals moved out of the winter aggregation area. No transmissions were received from a fourth tag until 39 days after deployment at which point the whale was south of the Western Australia and, although the tag transmitted for 22 days, there was insufficient data to interpolate a track suitable to be included in analyses. Of the nine tags deployed at Head of Bight (HOB), three tags failed to transmit, and three tags ceased transmitting within six days.

Migratory movements from coastal calving grounds were successfully obtained for six individuals (AI = 2, Pirates Bay (PB) = 1, HOB = 3). The six whales were tracked between 29 and 150 days with average individual distance per day of 20–61 km (Table 1). Although individual 96373 was tracked for 169 days no location was transmitted for 69 consecutive days.

**Table 1:** Details of satellite tag deployment duration and movement of six individuals for which migratory movements from coastal calving grounds were obtained. A state-space model framework was used to classify two behavioural modes based on mean turning angles and autocorrelation in speed and direction: travel and Area Restricted Search (ARS). Data on speed for each behavioural mode, and total duration of ARS behaviour for each individual whale are summarised below.

Tag ID	Age class—Sex	Deployment location	Deployment date	Duty cycle	Tracking duration (days)	Track distance (km)	Distance (km) / hour (mean ± sd; range)		Distance (km) / day (mean ± sd; range)	ARS behaviour duration (days) Estimated / Uncertain
							Travel	ARS		
96373	Adult Male	Auckland Islands, New Zealand	26/07/2009	6 hrs on 18 hrs off	100	2111	2.54 ± 1.34 [0.73–4.65]	0.62 ± 0.58 [0.01–3.12]	21 ± 22 [0.5–110]	85 / 1
96374	Adult Female	Auckland Islands, New Zealand	31/07/2009	6 hrs on 18 hrs off	150	5953	2.87 ± 1.61 [0.08–6.31]	0.63 ± 0.54 [0.01–3.18]	40 ± 38 [2–145]	67 / 20
98103	Sub-adult unknown	Pirates Bay, Tasmania	25/10/2010	4 hrs on 8 hrs off	103	6389	2.96 ± 1.31 [0.18–8.85]	0.87 ± 0.50 [0.14–2.55]	61 ± 34 [5–179]	12 / 6
120944	Adult female with calf	Head of Bight, South Australia	07/09/2014	3 hrs on 3 hrs off	29	1663	2.90 ± 1.67 [0.05–5.69]	1.48 ± 1.48 [0.07–6.35]	54 ± 41 [2–145]	2 / 10
120945	Adult female with calf	Head of Bight, South Australia	07/09/2014	3 hrs on 3 hrs off	57	1858	2.03 ± 1.70 [0.03–6.37]	0.29 ± 0.38 [0.01–1.55]	32 ± 36 [0.5–150]	19 / 4
120949	Adult female with calf	Head of Bight, South Australia	07/09/2014	3 hrs on 3 hrs off	32	645	4.93 ± 1.94 [1.65–7.73]	0.23 ± 0.34 [0.01–1.68]	20 ± 39 [0.4–163	26 / 1

**Fig 2:**
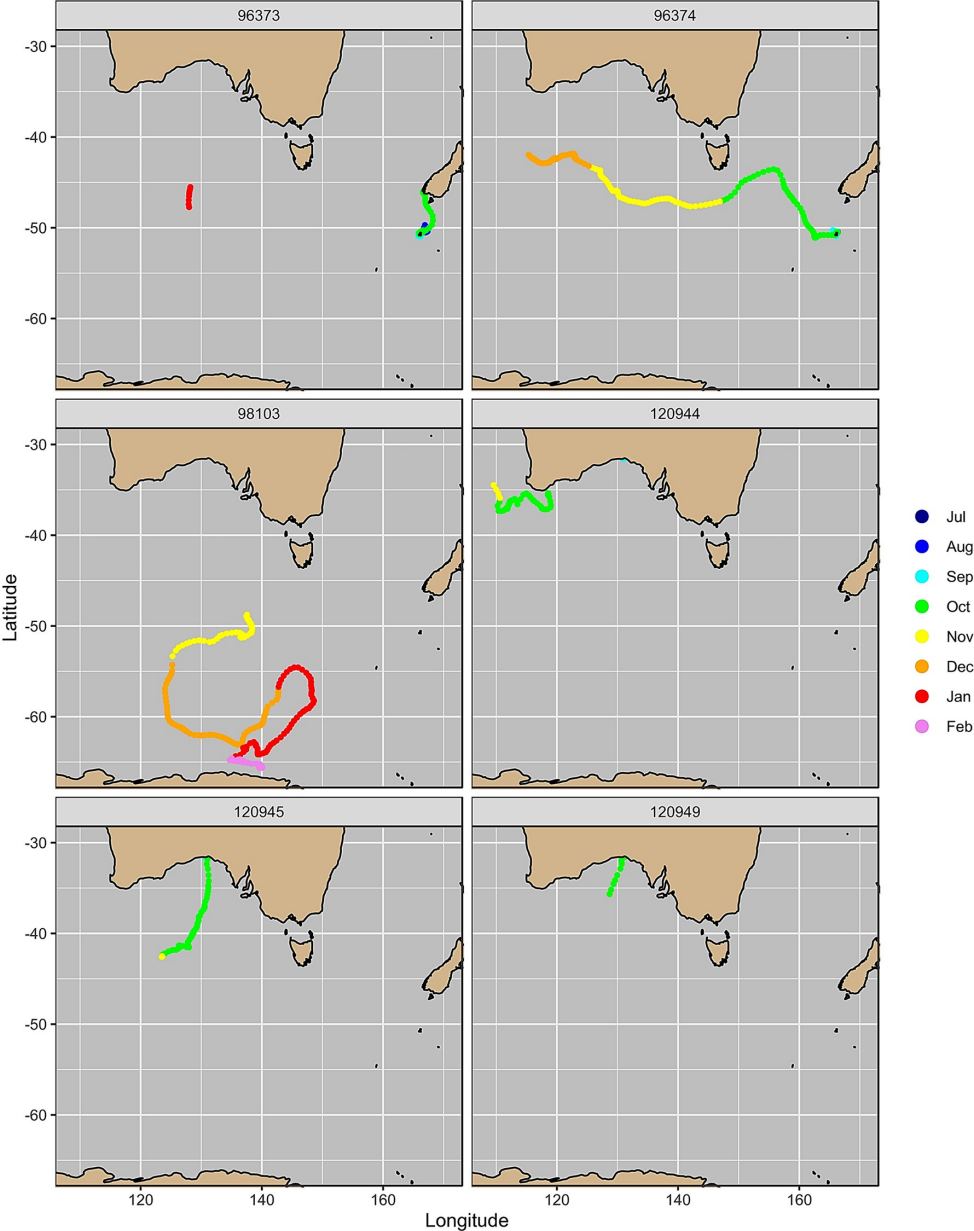
Interpolated satellite tracks of migrating southern right whales coloured by month. **Individuals 96373 and 96374 were tagged at the Auckland Islands, New Zealand.** Individual 98103 was tagged at Pirates Bay, Tasmania, Australia. Individuals 120944, 120945 and 120949 were tagged at Head of Bight, Australia.

Of the two whales tagged in July at the Auckland Islands for which there are data, both departed the aggregation area in October. The adult female (96374) migrated west and then northwest to approximately 43°S before moving westwards between 45–48°S, while the adult male (96373) initially migrated north to the South Island of New Zealand after which locations ceased to transmit until the individual was at approximately 126°E (Fig 2). The sub-adult whale tagged at Tasmania migrated southwest during October before moving in a more southerly direction around 55°S. During December, this individual migrated east then north-east returning back to approximately 55°S around the start of January, after which it began a second southwesterly migration until reaching 65°S at the beginning of February. Two of the individuals tagged at Head of Bight migrated southwest from the aggregation area in October. The tag on one individual (120949) ceased transmitting at 35°S in October, while the other individual (120945) continued to migrate south until 41°S after which its direction was more westerly. No location data were received for the third individual (120944) until early October at which stage the whale was approximately 1,320 km west of the HOB. This individual continued in a westerly migration between 35–38°S (Fig 2).

### Satellite tagging—Movement and foraging in relation to oceanographic features

Behavioural modes inferred by the model indicated that ARS occurred for 45% of the SSM locations. Transiting behaviour occurred for 46% of the SSM locations and uncertain foraging behaviour corresponded to 9% of the SSM locations. Search like behaviour persisted for 2 to 85 days depending on the individual (12 to 87 days if uncertain behaviour is included) (Table 1). This corresponds to 7 to 85% (18 to 86% if uncertain behaviour is included) of tracking duration.

**Fig 3:**
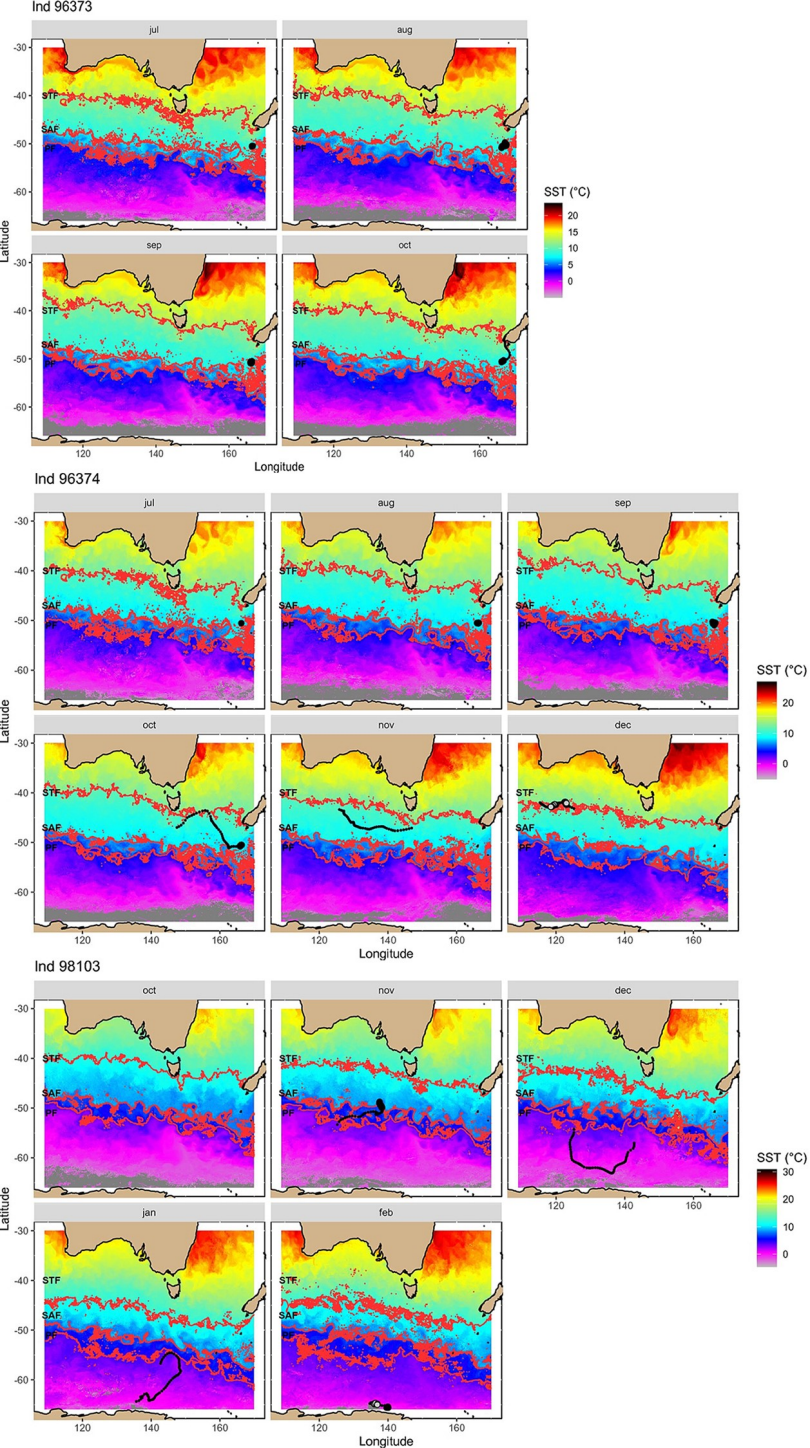
Interpolated movement tracks of tagged southern right whales from ARGOS location data plotted with sea surface temperature (SST) averaged over each month that the tag was transmitting. Individuals 96373 and 96374 were tagged at the Auckland Islands, New Zealand. Individual 98103 was tagged at Pirates Bay, Tasmania, Australia. Red dashed lines indicate the predicted location of the main oceanic fronts: Subtropical front (STF), sub-Antarctic front (SAF) and Polar Front (PF). Large black dots indicate areas of restricted search. Grey dots correspond to uncertain areas of restricted search inferred by the SSM.

**Fig 4:**
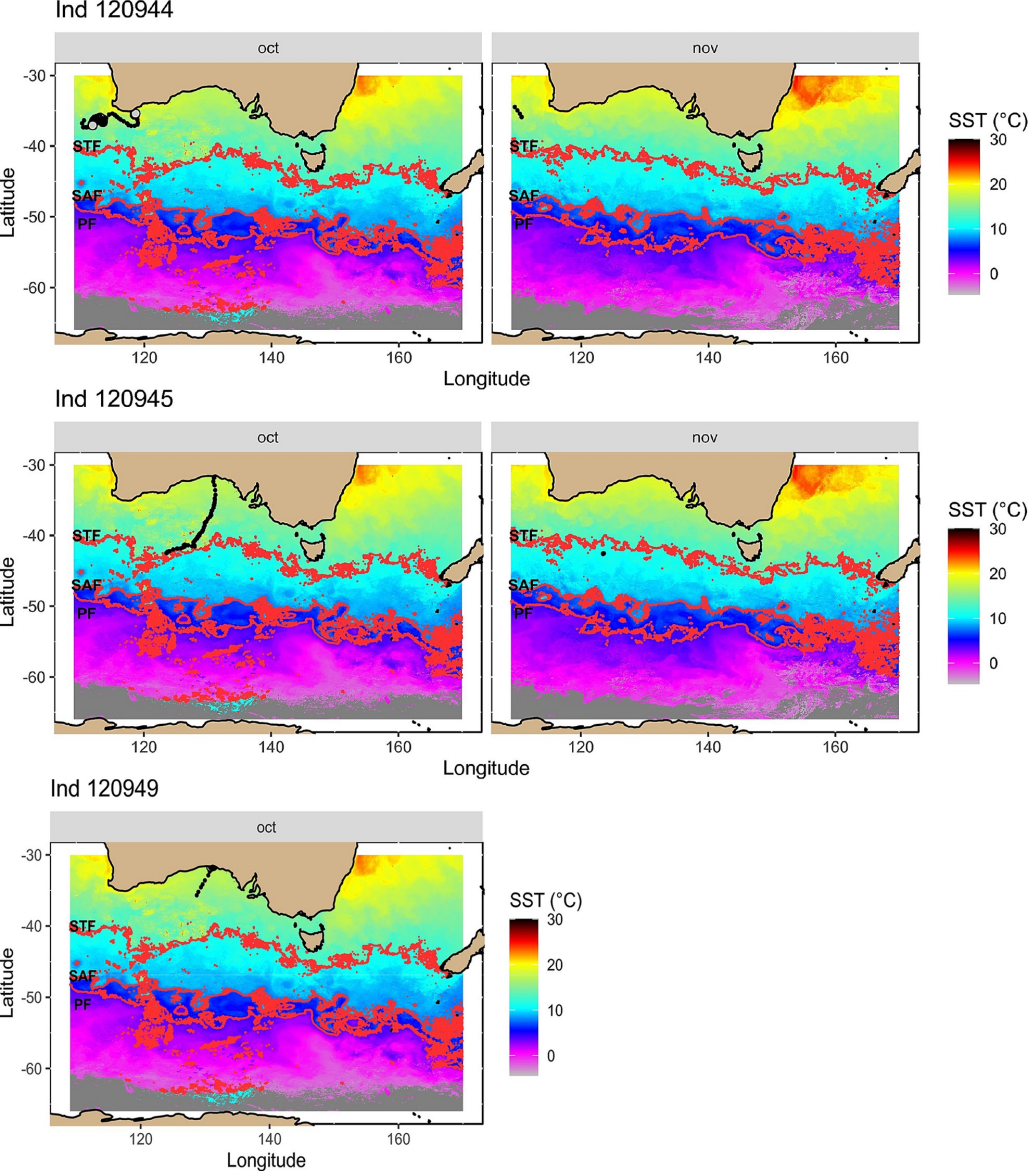
Interpolated movement tracks of tagged southern right whales tagged at Head of Bight, Australia, from ARGOS location data plotted with sea surface temperature (SST) averaged over each month that the tag was transmitting. Red dashed lines indicate the predicted location of the main oceanic fronts: Subtropical front (STF), sub-Antarctic front (SAF) and Polar Front (PF). Large black dots indicate areas of restricted search. Grey dots correspond to uncertain areas of restricted search inferred by the SSM.

Location data from the two whales tagged at the Auckland Islands Bight showed associations with the Subtropical Front (STF) (Figs 2 and 3). Individual 96373 was in the area of the STF off the southern coast of the south island of New Zealand in October, and individual 96374 associated with the area of the front in October and December. One whale tagged at Head of Bight (120145) moved in a mainly southward direction until it reached the area of the STF in October (Fig 4), at which point changed direction and moved westward within the estimated region of the front. The whale tagged in Tasmania migrated through the area of the the sub-Antarctic Front (SAF) in November and December (Figs 2 and 3). This individual headed towards Antarctica and then swam northward to reach the likely area of the Polar Front (PF) in January after which it returned south and was potentially off the ice edge when the tag stopped transmitting in February (Figs 2 and 3). In contrast, one whale from HOB (120144) stayed in warmers waters throughout October and until the tag ceased transmitting in November, and showed strong associations with areas of low sea level height and high current flow, which are indicative of low-pressure eddies that result in areas of localised upwelling (Fig 4). For five individuals, the results of SSM identified areas of increased residency times, potentially indicative of feeding, associated with the STF and SAF (Figs 3 and 4). The sixth remained at lower latitudes and SSM indicated increased residency time in association with low pressure eddies off the southwest of Australia (Fig 4).

**Fig 5:**
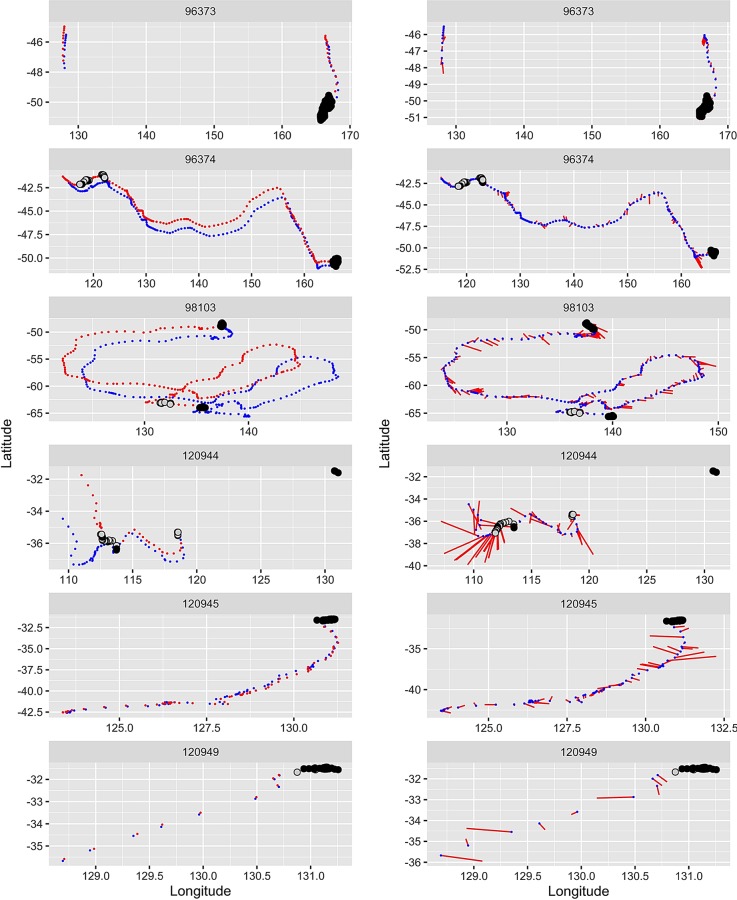
Left plots: Observed trajectories of southern right whales (blue dots) with superimposed surface current vectors (red). The black and grey dots indicate the estimated and uncertain foraging locations respectively. Right plots: Comparison of the observed (blue) and current-corrected (red) tracks. Foraging locations are positioned on the current-corrected track at the same dates as on the observed track. Note that the current-corrected track displays the animal’s own motion integrated in time but, taken alone, a position along that track bears no direct interpretation.

The comparison of the observed and current-corrected tracks (Fig 5) indicates that in general, currents did not affect the travel trajectories of the tagged southern right whales. However, on some occasions, especially for individuals 120944 (HOB) and 98103 (PB), their trajectories were strongly modified by current during some foraging phases. The observed trajectory of 120944 appears to be circular at the end of the track, with initial movement southwards. However, when the trajectory is corrected for current, the track indicates the individual was actually moving north, which suggests the whale was facing into the direction of the current and either drifting passively, or moving slowly, during that section of the track. The trajectory of the sub-adult whale tagged in Tasmania (98103) also shows a slight variation in trajectory when the track is corrected for current. While the observed trajectory suddenly heads south-east at the beginning of the track, the individual continues to follow its initial movement direction, suggesting the whale was either drifting passively or moving slowly facing into the current during this section of the track.

## Discussion

This study presents the first contemporary data on the migratory movements of southern right whales from wintering grounds in Australasia, providing new information on migratory corridors and identifying areas of restricted search and increased residency, which could be indicative of feeding. These data identify four potential foraging grounds for southern right whales that calve and/or winter in Australia and New Zealand: south-west Western Australia, Subtropical Front (STF), sub-Antarctic front (SAF) and Polar Front (PF). The use of multiple feeding grounds by individuals from a single wintering ground has also been found for South Atlantic southern right whales satellite tracked from wintering grounds [28–29, 48] and inferred from stable isotope data [6–7,25].

Latitudes associated with the STF (39°–42°S) appear to be a feeding ground for both New Zealand and Australian southern right whales. The two southern right whales tagged at the Auckland Islands (96373, 96374), both moved westwards within the region of the STF, with one of the individuals initially moved northwards from the Auckland Islands to the area of the STF south of the New Zealand mainland prior to its westward migration. One of the individuals (120945) tagged at Head of Bight also migrated to the region of the STF and a second individual showed similar southerly movement before its tag stopped transmitting. Historical whaling data show catches of southern right whales occurred in the region of the STF south of Australia in the Austral summer months [30], and two photo-ID matches have been made between southern right whales photographed around 43°S in December 1995, and the southwest Australian wintering grounds [49]. One whale had previously been photographed in South Australia in June 1994, the other off Western Australia in September 1995 [49]. The STF lies within the Southern Tropical Convergence (STC) Three outhern right whales satellite tagged in South Africa also showed an association with the STC in the South Atlantic during the Austral summer months ([28], and recent data from southern right whales tagged off Península Valdéz, Argentina, showed migratory movement of one female accompanied by a calf to latitudes associated with the STC [29]. Based on historical whaling data [30] and more recent telemetry studies, the STF south of Australia and New Zealand is suggested to be an important feeding for a number of other baleen whale species including pygmy blue whales [39] and humpback whales [50].

In contrast, one individual (120944) tagged at Head of Bight was associated with a low-pressure cyclonic eddy off the south-west tip of Western Australia. These quasi-periodic eddies occur annually in the region during the Austral summer and are driven by the interaction between a weakening in the southerly flowing Leeuwin Current and persistent seasonal southerly winds that results in localised upwelling [51]. Results of the state-space model identified higher time spent in a restricted area in this part of the individual’s track. Similar associations between a southern right whale satellite tagged in Argentina and cyclonic eddies have been observed [29]. While information is limited on southern right whale feeding behaviour, North Atlantic right whales undertake surface feeding when copepods are aggregated in patches [52] Correcting the track trajectory of this individual for current speed suggests that the individual turned to face into the current flow and either drifted or moved slowly into the current, potentially surface feeding. The last location received for 120944 was from the southwest edge of the Naturaliste Plateau, an area where whaling was undertaken historically in the Austral summer [30]. This region was also found to be an area of high occupancy by satellite tagged pygmy blue whales prior to their northward migration up the western coast of Australia [39]. As satellite location data were not transmitted for this individual until it was south of Albany, Western Australia, it is unknown when the whale departed Head of Bight or the route it took moving westwards.

The sub-adult whale tagged at Pirates Bay Tasmania, showed more variation in direction of travel than the other five tagged right whales, moving between latitudes 55°S and 65°S. This individual was potentially in the vicinity of the ice edge when the tag ceased transmitting. Previously, the only matches from Australia to Antarctica was one whale matched via photo-ID between Western Australia (~700 whales in the catalogue at the time) and south of 60° S (19 photo-IDs collected: [53]), although this match was directly south of Western Australia and further west than the track of the Tasmanian whale.

These data show the variability in migratory behaviour by individuals from Australia and New Zealand wintering grounds and is consistent with findings of within ocean basin differences in foraging areas used by southern right whales from populations in South Africa and Argentina [28–29, 48]. As data were collected over six years from three different migrations it is possible that the differences in movement patterns observed simply reflect prey availability and distribution during the period that each individual whale was tagged. Isotopic data show that some right whales have mixed foraging strategies [25], and individual southern right whales satellite tagged in Argentina were found to move between potential foraging areas within the same season [48]. This variability in foraging strategies is also supported by whaling data, where stomach contents of southern right whales harvested at latitudes below 40°S were dominated by copepods, whilst those taken above 50°S were dominated by krill, with a mixture of both prey items in stomachs from animals taken from intervening latitudes [54]. North Atlantic right whales also show mixed foraging strategies and individual migratory flexibility, and movements between foraging grounds within a season are likely driven by environmental conditions as well as the age and sex of individuals [55, 56]. The only sub-adult in the current study was the individual tagged in Tasmania.

Based on historical whaling data and contemporary tracking data from southern right whale populations in South Africa and Argentina, the areas of restricted movement identified in this study were assumed to most likely indicate feeding behaviour. However, it is also possible that such areas of increased residency were a result of whales being engaged in other behaviours such as resting or socialising.

### Implications for population structure and recovery

Comparison of individual southern right whale stable isotope and genetic profiles indicate maternally directed site fidelity from calving areas in Argentina and Australia to specific feeding areas [6, 7]. Migratory fidelity to specific feeding and wintering grounds has been attributed as the causative driver of genetic structuring in Australian and New Zealand southern right whales across their migratory network [7]. However, while southern right whales generally show fidelity to a particular calving ground, the genetic match of two female whales biopsied at the Head of Bight to whales previously sampled in the Auckland Islands in the current study (see S1 Data) supports previous observations of some limited interchange of females between these two calving grounds [57]. Evidence of movements of females between calving grounds in the South Atlantic have also been observed [10,58]. The two females sampled in both HOB and in the Auckland Islands were only seen once in New Zealand waters, and were associated with calves only in the Head of Bight wintering aggregation. This suggests these could be exploratory or temporary immigration events to New Zealand from Australia, although extrapolation from such a small sample size remains speculative. The fact that the wintering ground matches are between regions that appear to share foraging grounds, albeit in different years, is consistent with the hypothesis that shared feeding grounds facilitates connectivity between wintering grounds [8].

It is striking that the single whale tagged in SEA showed a movement pattern distinct from both the HOB and New Zealand whales, potentially reflecting a different foraging strategy or a different migratory behaviour for juvenile whales. The sub-adult tagged at Tasmania moved directly through the area of the STF, where HOB and NZ right whales were likely to be foraging, to much higher latitudes where it remained until the tag ceased transmitting. Environmental conditions at foraging grounds, a proxy for prey abundance, are highly correlated with reproductive success in southern [26, 27] and North Atlantic right whale wintering grounds [59].

The comparatively slow recovery of the southeast Australian and mainland New Zealand wintering aggregations has been hypothesised to be due to high historical hunting pressure on the former, and the potential that cultural memory of calving grounds was lost, resulting in slow recolonisation of these areas [18, 60]. It may also be due to contemporary unsuitability of wintering habitats as a result of increased anthropogenic disturbance in these areas, or may reflect maternal fidelity to sub-optimal foraging areas leading to limitations in population growth. It is not possible to conclude whether there are inherent differences in migratory routes and foraging areas between the SWA and SEA populations from the movement of a single sub-adult whale tagged in Tasmania. Therefore further data on movement and foraging ground preferences of whales from wintering grounds that show different recovery trajectories are required to understand whether there is a link between foraging areas and sub-population trajectories.

### Limitations

While satellite telemetry has provided important data for the conservation and management of large whale species, the use of implantable tags has raised several concerns with respect to possible short and/or long-term adverse effects of these devices on tagged individuals [61, 62], and so it is critical that the information to be gained from studies is carefully weighed against potential impacts and alternative methods are considered [63]. The use of implantable satellite tags was required to provide the baseline information needed to assess potential threats to Australasian southern right whales during migration and at foraging grounds.

Poor tag transmission meant that 10 of 16 deployed satellite tags failed to provide information on the offshore migratory movement of southern right whales. Of these 10, six ceased transmitting before migration began, three failed to transmit post implantation and one did not transmit consistently enough to be included in analysis. Failure to transmit or variability in transmission performance may have been caused by mechanical or electronic failure [61], poor implantation and subsequent shedding of the tag [29, 64], or sub-optimal position of tag deployment. Two of the nine satellite tags deployed at HOB failed to implant properly, while one which implanted properly is suspected to have failed electronically. In order to obtain offshore movement patterns from HOB, satellite tagging was conducted at the end of the aggregation period at HOB, when most remaining individuals were females with calves. It is very possible that high tag failure in this study was due to tag damage as a result of the calf’s thigmotactic behaviour [28, 65], and tag failure would have been reduced if more unaccompanied adults had been available.

### Management implications

In contrast to some baleen whale species that show movement along migratory corridors, southern right whales from Australia and New Zealand show more diffuse offshore movement patterns as well as differences in potential Austral summer foraging grounds. These individual variations show that potential anthropogenic impacts on these populations need to be considered throughout their distribution range both within and outside of territorial waters. North Atlantic right whale also show diffuse migration and individuals have been recorded year round in what were previously considered migratory corridors [66], and analyses of long-term acoustic and visual data shown large scale distribution of the species as well as evidence that not all the population undergo annual migrations [67].

As southern right whale populations continue to recover across Australia and New Zealand wintering grounds, the likelihood for interactions with anthropogenic activities such as shipping, seismic surveys, fisheries and coastal development is expected to increase [7, 10, 68]. Sub-lethal impacts of vessel noise include chronic stress [69]), and changes in vocalisation behaviour [70]). The cumulative effect of sub-lethal impacts is now recognised as a threat to the persistence of whale populations. For example, the impact of non-lethal entanglement on energetic costs [71,72], stress [73–75] and reproductive output of individual whales has been quantified in the North Atlantic right whale. Future studies should continue to investigate and quantify what proportion of time right whales spend in highly modified coastal areas where they may be exposed to lethal and sub-lethal threats, compared with their more remote and less modified feeding areas.

We identified four potential summer foraging grounds, with one, the area of the STF being visited by whales from both the Head of Bight and Auckland Island calving areas. Given the small sample size, further data would be required to determine how representative these potential foraging grounds are and the level of fidelity, or otherwise, to these areas from different populations. However, fidelity to different quality feeding grounds may be one factor leading to observed differences in recovery rates of sub-population in Australasia and information on the location of foraging grounds is essential to understand the impacts that future ecosystem change may have on different populations.

## Supporting information

S1 Data (DOCX)
