# Correction: Satellite derived offshore migratory movements of southern right whales (*Eubalaena australis*) from Australian and New Zealand wintering grounds

**DOI:** 10.1371/journal.pone.0235186

**Published:** 2020-06-18

**Authors:** Alice I. Mackay, Frédéric Bailleul, Emma L. Carroll, Virginia Andrews-Goff, C. Scott Baker, John Bannister, Laura Boren, Kris Carlyon, David M. Donnelly, Michael Double, Simon D. Goldsworthy, Robert Harcourt, Dirk Holman, Andrew Lowther, Guido J. Parra, Simon J. Childerhouse

The eighth author's name is spelled incorrectly. The correct name is: Kris Carlyon.
