# Supplementary material for: Satellite derived offshore migratory movements of southern right whales (Eubalaena australis) from Australian and New Zealand wintering grounds
Source: PLoS One. 2020 May 7;15(5):e0231577. doi: 10.1371/journal.pone.0231577 (PMC7205476; doi:10.1371/journal.pone.0231577)
Supplement: S1 Data — (DOCX) [file pone.0231577.s001.docx]

# Supporting information – genetic data

## Biopsy collection

Where possible, biopsy samples were collected concurrently to tag deployment using a biopsy dart fired from a modified veterinary capture device [1]. At the Head of the Bight and the Auckland Islands, additional samples were collected wherever possible from whales not the focus of tagging. Skin samples were preserved in 70% ethanol on location and transferred to the University of St Andrews for curation and storage at -20C. DNA was extracted from skin samples using a standard proteinase K digestion and phenol/chloroform method [2]. DNA profiles were constructed for each sample, comprising mitochondrial DNA (mtDNA) control region haplotype (500 bp), sex and up to 17 microsatellite loci. Molecular identification of sex and sequencing of the mtDNA control region follow methods previously described [3]. Microsatellite genotyping at up to 17 loci (EV1, EV14, EV37, EV94: [4]; GATA28, GATA98: [5]; RW31, RW410, RW18, RW48: [6]; CA232, GT122, GT23, GT310: [7]; TR3G1, TR3G2, TR3F4:[8] following previously published methodology [9]. To ensure consistent identification between this and previous studies, a set of six New Zealand samples were amplified and run alongside the Head of Bight samples. Alleles were sized with Genemapper v4.0 (Applied Biosystems) and all automated calling was confirmed by eye [10]. These loci have previously been used in the New Zealand and Australian population without evidence of significant deviation from Hardy-Weinberg equilibrium [9], so we do not repeat these analyses here.

## Genotype matching

We used CERVUS v3.0 [11], to identify repeat sampling of the same genotype, assumed to be the same individual whale, within the Head of Bight dataset. Confidence in matches were given by estimating the probability of identity [12], calculated in CERVUS. As a precaution against false exclusion due to genotyping errors, the initial comparison allowed for mismatches at up to three loci [13]. The electropherograms of the mismatching loci were then reviewed and either corrected based on this visual inspection or repeated.

Using the same methodology, we also compared the unique genotypes from the Head of Bight dataset to the New Zealand and Australian southern right whale genotype catalogues. The New Zealand catalogue comprises 692 whales sampled in the Auckland Islands [14] and 47 whales sampled around the North and South Islands (mainland) of New Zealand (6 of which were also seen in the Auckland Islands. The Australian catalogue comprises 78 whales sampled across the species’ distribution, from Queensland to Western Australia [9]. Matches and Probability of Identity (PID) were calculated based on the 13 microsatellite loci used across the genotype catalogues as only a relatively small subset of the New Zealand dataset has been genotyped for the full 17 microsatellite loci.

## Results

The 19 skin biopsy samples collected during Head of Bight field work were successfully genotyped at an average of 16.8 of 17 loci (Table S1). Comparison of DNA profiles amongst the 19 samples revealed 16 unique whales had been sampled. One whale had been sampled twice and one thrice, with probability of identities showing a very low chance of the same genotype occurring by chance (8.07E-21 and 4.87E-17, respectively: see Appendix GENOTYPE Table 2 for match details).

Comparison of the unique Head of Bight samples with the Australian and New Zealand genotype catalogues revealed two matches, both back to the Auckland Islands. The matches were to females only seen in the Auckland Islands once (1995 and 2006), both of which were seen without calves in New Zealand waters (Table S2). One of these matches was from a whale that was satellite tagged at the Head of Bight (Eau14HOB01, tag 112275), however, this tag did not provide many location fixes and so was excluded from overall analysis of tracking data.

S1: DNA profiles of whales sampled at Head of Bight, South Australia: mtDNA control region haplotype (500 bp; mtDNA), genetically identified sex and microsatellite genotype. Dashed lines indicate the sample was not successfully genotyped at that locus. Rep denotes the within-season match to another sample and tag indicates satellite tag number.

| Sample | Status | Sex | mtDNA | Rep | Tag | EV1 | EV14 | EV37 | GATA28 | GATA98 | GT23 | RW18 | RW31 | RW410 | RW48 |
| --- | --- | --- | --- | --- | --- | --- | --- | --- | --- | --- | --- | --- | --- | --- | --- |
| Eau14HOB01 | cow | F | BAKHAPB+ |  | 112725 | 122/140 | 133/133 | 195/197 | 166/178 | 112/116 | 108/118 | 193/193 | 123/125 | 197/203 | 108/122 |
| Eau14HOB02 | cow | F | BAKHAPA |  | 112729 | 124/142 | 129/133 | 203/207 | 174/174 | 108/116 | 118/120 | 189/195 | 121/123 | 195/205 | 108/120 |
| Eau14HOB03 | cow | F | BAKHAPD |  | 121199 | 122/122 | 133/141 | 189/193 | 166/170 | 116/120 | 114/114 | 189/195 | 125/125 | 191/195 | 118/120 |
| Eau14HOB04 | cow | F | BAKHAPB+ |  | 121209 | 126/128 | 131/141 | 187/201 | 170/182 | 116/116 | 110/118 | 189/195 | 123/127 | 195/199 | 118/120 |
| Eau14HOB05 | cow | F | BAKHAPA |  | 120945 | 122/142 | 133/133 | 199/207 | 166/178 | 104/116 | 116/116 | 193/199 | 123/125 | 195/211 | 120/122 |
| Eau14HOB06 | cow | F | BakHapB+ |  | 120949 | 122/142 | 129/131 | 197/203 | 166/170 | 108/116 | 114/118 | 187/193 | 125/125 | 197/203 | 120/122 |
| Eau14HOB07 | cow | F | BAKHAPA |  |  | 122/122 | 131/133 | 189/193 | 166/174 | 112/116 | 110/120 | 193/193 | 123/125 | 191/205 | 120/124 |
| Eau14HOB08 | cow | F | BAKHAPE |  |  | 122/134 | 133/141 | 193/195 | 166/178 | 112/116 | 114/114 | 187/193 | 121/123 | 195/205 | 124/124 |
| Eau14HOB09 | cow | F | BAKHAPA | Eau14HOB16 |  | 122/158 | 133/137 | 193/195 | 178/178 | 104/116 | 112/116 | 193/193 | 119/127 | 199/203 | 118/120 |
| Eau14HOB10 | cow | F | BAKHAPB' |  |  | 134/148 | 133/133 | 193/201 | 166/166 | 108/116 | 112/114 | 193/193 | 123/125 | 195/205 | 108/126 |
| Eau14HOB11 | cow | F | BakHapB+ |  |  | 130/142 | 122/133 | 197/201 | 174/178 | 112/116 | 112/116 | 187/231 | 121/123 | 199/209 | 118/126 |
| Eau14HOB12 | cow | F | BAKHAPB' | Eau14HOB15 & 13 |  | 126/148 | 141/141 | 199/203 | 162/166 | 112/116 | 114/116 | 193/199 | 117/117 | 195/205 | 118/126 |
| Eau14HOB14 | cow | F | BAKHAPA |  |  | 126/138 | 141/141 | 193/201 | 166/178 | 108/108 | 112/118 | 189/193 | 121/123 | 197/209 | 122/124 |
| Eau14HOB17 | cow | F | BAKHAPA |  |  | 130/132 | 137/137 | 193/203 | 166/178 | 108/112 | 118/120 | 187/189 | 123/125 | 197/197 | 122/124 |
| Eau14HOB18 | cow | F | BAKHAPD |  |  | 148/158 | 137/139 | 189/201 | 166/178 | 112/120 | 120/120 | 189/199 | 121/123 | 207/211 | 120/126 |
| Eau14HOB19 | cow | F | BAKHAPA |  |  | 126/146 | 133/135 | 193/203 | 174/178 | 116/116 | 112/116 | 195/215 | 123/123 | 197/205 | 122/122 |

| Sample | TR3F4 | TR3G1 | TR3G2 | CA232 | EV94 | GT310 |  |  |  |  |
| --- | --- | --- | --- | --- | --- | --- | --- | --- | --- | --- |
| Eau14HOB01 | 305/313 | 222/222 | 176/176 | 140/140 | 196/196 | 96/102 |  |  |  |  |
| Eau14HOB02 | 317/353 | 206/242 | 176/180 | 148/148 | 196/200 | 96/102 |  |  |  |  |
| Eau14HOB03 | 305/309 | 210/210 | 172/172 | 140/148 | 196/196 | 100/100 |  |  |  |  |
| Eau14HOB04 | 305/317 | 222/222 | 172/184 | 146/148 | 196/196 | 98/100 |  |  |  |  |
| Eau14HOB05 | 301/301 | 218/238 | 176/184 | 140/140 | 196/196 | 96/98 |  |  |  |  |
| Eau14HOB06 | 301/309 | 206/210 | 172/176 | 146/148 | 196/196 | 96/98 |  |  |  |  |
| Eau14HOB07 | 305/309 | 206/210 | 176/180 | 140/140 | 196/200 | -/- |  |  |  |  |
| Eau14HOB08 | 305/345 | 214/234 | 184/184 | 140/150 | 198/200 | 96/100 |  |  |  |  |
| Eau14HOB09 | 309/321 | 206/210 | 168/172 | 140/142 | 196/196 | -/- |  |  |  |  |
| Eau14HOB10 | 309/317 | 214/238 | 168/180 | 142/142 | 196/196 | 96/100 |  |  |  |  |
| Eau14HOB11 | 301/305 | 222/222 | 176/184 | 142/148 | 196/196 | 96/100 |  |  |  |  |
| Eau14HOB12 | 313/321 | 206/238 | 172/184 | 140/148 | 196/200 | 100/102 |  |  |  |  |
| Eau14HOB14 | 333/337 | 206/234 | 176/184 | 138/140 | 196/196 | 100/100 |  |  |  |  |
| Eau14HOB17 | 309/333 | 222/234 | 176/184 | 140/142 | 196/200 | 100/100 |  |  |  |  |
| Eau14HOB18 | 301/317 | 210/234 | 172/184 | 140/140 | 196/200 | 100/100 |  |  |  |  |
| Eau14HOB19 | 301/333 | 206/206 | 180/184 | 142/148 | 196/196 | 100/102 |  |  |  |  |

S22: DNA profiles thought to be the same individuals, based on number of matching loci (Match) and probability of identity (PID), based on the first 13 microsatellite loci in the table (for which all New Zealand samples are typed at). DNA profiles comprise: mtDNA control region haplotype (500 bp; mtDNA), genetically identified sex and microsatellite genotype. Dashed lines indicate the sample was not successfully genotyped at that locus.

| Sample | Status | Sex | mtDNA | Sampling location | Year | Match/  PID | EV1 | EV14 | EV37 | GATA28 | GATA98 | GT23 | RW18 | RW31 | RW410 | RW48 |
| --- | --- | --- | --- | --- | --- | --- | --- | --- | --- | --- | --- | --- | --- | --- | --- | --- |
| Eau14HOB09 | cow | F | BAKHAPA | Head of Bight | 2014 | 13/ | 122/158 | 133/137 | 193/195 | 178/178 | 104/116 | 112/116 | 193/193 | 119/127 | 199/203 | 118/120 |
| Eau14HOB16 | cow | F | BAKHAPA | Head of Bight | 2014 | 8.07E-21 | 122/158 | 133/137 | 193/195 | 178/178 | 104/116 | 112/116 | 193/193 | 119/127 | 199/203 | 118/120 |
| Eau06AI005 | adult | F | BAKHAPA | Auckland Is. | 2006 |  | 122/158 | 133/137 | 193/195 | 178/178 | 104/116 | 112/116 | 193/193 | 119/127 | 199/203 | 118/120 |
| Eau14HOB12 | cow | F | BAKHAPB' | Head of Bight | 2014 | 12-13/  4.87E-19 - 4.69E19 | 126/148 | 141/141 | 199/203 | 162/166 | 112/116 | 114/116 | 193/199 | 117/117 | 195/205 | 118/126 |
| Eau14HOB13 | cow | F | fail | Head of Bight | 2014 |  | 126/148 | 141/141 | 199/203 | 162/166 | 112/116 | 114/116 | 193/199 | 117/117 | 195/205 | 118/126 |
| Eau14HOB15 | cow | F | BAKHAPB' | Head of Bight | 2014 |  | 126/148 | -/- | 199/203 | 162/166 | 112/116 | 114/116 | 193/199 | 117/117 | 195/205 | 118/126 |
| Eau14HOB01 | cow | F | BAKHAPB+ | Head of Bight | 2014 | 10/ | 122/140 | 133/133 | 195/197 | 166/178 | 112/116 | 108/118 | 193/193 | 123/125 | 197/203 | 108/122 |
| Eau95AI055 | adult | F | BAKHAPB+ | Auckland Is. | 1995 | 3.21E-14 | 122/140 | -/- | 195/197 | 166/178 | 112/116 | 108/118 | 193/193 | 123/125 | 197/203 | -/- |

| Sample | TR3F4 | TR3G1 | TR3G2 | CA232 | EV94 | GT310 |  |
| --- | --- | --- | --- | --- | --- | --- | --- |
| Eau14HOB09 | 309/321 | 206/210 | 168/172 | 140/142 | 196/196 | -/- |  |
| Eau14HOB16 | 309/321 | 206/210 | 168/172 | 140/142 | 196/196 | 96/100 |  |
| Eau06AI005 | 309/321 | 206/210 | 168/172 | Not genotyped at these loci | | | |
| Eau14HOB12 | 313/321 | 206/238 | 172/184 | 140/148 | 196/200 | 100/102 |  |
| Eau14HOB13 | 313/321 | 206/238 | 172/184 | 140/148 | 196/200 | 100/102 |  |
| Eau14HOB15 | 313/321 | 206/238 | 172/184 | 140/148 | 196/200 | -/- |  |
| Eau14HOB01 | 305/313 | 222/222 | 176/176 | 140/140 | 196/196 | 96/102 |  |
| Eau95AI055 | 305/313 | -/- | 176/176 | Not typed at these loci | | | |

## References

1. Krützen M, Barré LM, Möller LM, Heithaus MR, Simms C, Sherwin WB. A biopsy system for small cetaceans: darting success and wound healing in Tursiops spp. Marine Mammal Science. 2002 Oct;18(4):863-78.
2. Sambrook J, Fritsch EF, Maniatis T (1989) Molecular cloning:a laboratory manual, 2nd edn. Cold Spring Harbor Labo-ratory Press, Cold Spring Harbor, NY
3. Carroll E, Patenaude N, Alexander A, Steel D, Harcourt R, Childerhouse S, Smith S, Bannister J, Constantine R, Baker CS. Population structure and individual movement of southern right whales around New Zealand and Australia. Marine Ecology Progress Series. 2011 Jun 27;432:257-68.
4. Valsecchi E, Amos W. Microsatellite markers for the study of cetacean populations. Molecular Ecology. 1996 Feb;5(1):151-6.
5. Palsbøll PJ, Bérubé M, Larsen AH, Jørgensen H. Primers for the amplification of tri‐and tetramer microsatellite loci in baleen whales. Molecular Ecology. 1997 Sep;6(9):893-5.
6. Waldick RC, Brown MW, White BN. Characterization and isolation of microsatellite loci from the endangered North Atlantic right whale. Molecular Ecology. 1999 Oct;8(10):1763-5.
7. Bérubé M, JØrgensen H, McEwing R, Palsbøll PJ. Polymorphic di‐nucleotide microsatellite loci isolated from the humpback whale, Megaptera novaeangliae. Molecular Ecology. 2000 Dec;9(12):2181-3.
8. Frasier TR, Rastogi T, Brown MW, Hamilton PK, Kraus SD, White BN. Characterization of tetranucleotide microsatellite loci and development and validation of multiplex reactions for the study of right whale species (genus Eubalaena). Molecular Ecology Notes. 2006 Dec;6(4):1025-9.
9. Carroll EL, Baker CS, Watson M, Alderman R, Bannister J, Gaggiotti OE, Gröcke DR, Patenaude N, Harcourt R. Cultural traditions across a migratory network shape the genetic structure of southern right whales around Australia and New Zealand. Scientific reports. 2015 Nov 9;5:16182.
10. Bonin A, Bellemain E, Bronken Eidesen P, Pompanon F, Brochmann C, Taberlet P. How to track and assess genotyping errors in population genetics studies. Molecular ecology. 2004 Nov;13(11):3261-73.
11. Kalinowski ST, Taper ML, Marshall TC. Revising how the computer program CERVUS accommodates genotyping error increases success in paternity assignment. Molecular ecology. 2007 Mar;16(5):1099-106.
12. Paetkau D, Strobeck C. Microsatellite analysis of genetic variation in black bear populations. Molecular ecology. 1994 Oct;3(5):489-95.
13. Waits JL, Leberg PL. Biases associated with population estimation using molecular tagging. In Animal Conservation forum 2000 Aug (Vol. 3, No. 3, pp. 191-199). Cambridge University Press.
14. Carroll EL, Childerhouse SJ, Fewster RM, Patenaude NJ, Steel D, Dunshea G, Boren L, Baker CS. Accounting for female reproductive cycles in a superpopulation capture–recapture framework. Ecological Applications. 2013 Oct;23(7):1677-90.
